# Temperature effect on extracellular polymeric substances (EPS) and phosphorus accumulating organisms (PAOs) for phosphorus release of anaerobic sludge†

**DOI:** 10.1039/c8ra10048a

**Published:** 2019-01-16

**Authors:** Fanzhe Zeng, Wenbiao Jin, Qingliang Zhao

**Affiliations:** State Key Laboratory of Urban Water Resources and Environment (SKLUWRE), School of Environment, Harbin Institute of Technology Harbin 150090 China qlzhao@hit.edu.cn +86-45186283017; School of Civil & Environmental Engineering, Harbin Institute of Technology Shenzhen 518055 China

## Abstract

Phosphorus (P) is an essential element for living organisms and anaerobic sludge is an attractive source for P recovery. Anaerobic P release depends on both phosphorus-accumulating organisms (PAOs) and extracellular polymeric substances (EPS). However, the P release contributed by the microbial cells and EPS was not addressed completely and the effect of temperature on the mechanism of P release and transformation was rarely considered. This study, therefore, investigated the effects of temperature on the P fraction and the relationship between PAOs metabolic pathway and EPS reaction using the Standards in Measurements and Testing (SMT) protocol and the ^31^P nuclear magnetic resonance (^31^P-NMR) experiments. Experimental results showed that the temperature not only affected the metabolism of PAOs, but also significantly influenced the EPS components and the hydrolysis of EPS-associated polyphosphate (poly-P). And the P release mainly occurred due to biological mechanisms with a conversion from non-reactive P (NRP) in both intracellular and extracellular substances to reactive P (RP) fractions. The highest concentration of total P in the supernatant (TP_L_) occurred at 15 °C, and the TP_L_ release from the solid to liquid phase was better fitted with pseudo-second-order kinetic model. More organic P in the sludge (OP_s_) released from the sludge phase at 35 °C would convert into inorganic P (IP_s_) and non-apatite inorganic phosphorus (NAIP_s_) was the most labile P fraction for P release. The hydrolysis of EPS-associated poly-P was enhanced by higher temperatures with the degradation of the long-chain poly-P by PAOs. Meanwhile, a lower temperature could obviously improve the P release because the dominance of PAOs would potentially shift to GAOs with the increase of temperature. But the very-low temperature (5 °C) was not beneficial for the P release and suppressed the microbial activities.

## Abstract 2

Phosphorus (P) is an essential element for living organisms and anaerobic sludge is an attractive source for P recovery.

## Graphical abstract

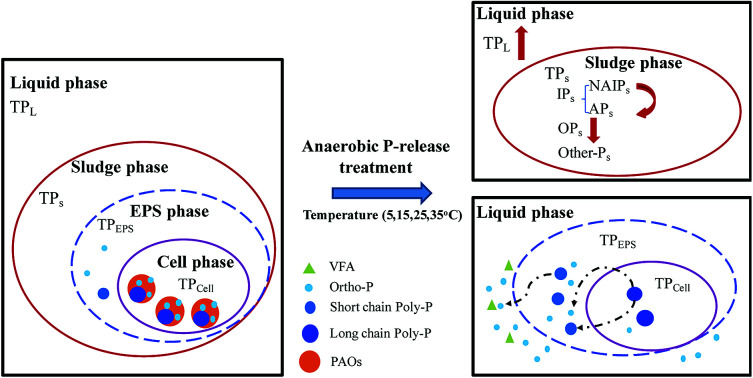

## 1. Introduction

Phosphorus (P) is an indispensable and non-renewable nutrient for agricultural fertilizers, but phosphate rocks will be exhausted in 100 years.^1^ Thus, P resource recovery is essential for a sustainable development of agriculture and society. The production of waste activated sludge (WAS) from wastewater treatment plants (WWTPs) increases rapidly with the development of urbanization and industrialization and meanwhile, is considered as a potential bioenergy source and hence can be used as a renewable energy production.^2^ More than 90% P flowing into the wastewater is accumulated in the sewage sludge during wastewater treatments. Sewage sludge contains more than 12% P of dry sludge by weight,^3,4^ and more than 40% of P exists in bounded biological biomass, while the rest is inorganic P of WAS.^5,6^ Thus, in order to use WAS as a P resource, the first step is to release P from WAS as more as possible for the following treatments of P recovery. According to sustainability policies and economic needs, the energy-efficient and environmentally friendly technology of phosphorus release from WAS is necessary and essential for WWTPs. Compared with chemical treatments, anaerobic phosphorus release from sewage sludge is verified as a sustainable and efficient P recovery method because of the avoidance of the eutrophication and algal bloom problems.^7^

The fraction of phosphorus in the sludge is important as not all kinds of phosphorus fraction exhibit bio-availability. Therefore, the phosphorus fraction will be selected to evaluate the performance of the phosphorus release behavior rather than total phosphorus (TP) concentration. It was also reported that 40–60% of reactive P (RP) was existed in sewage sludge, which could be recoverable and available for chemical reactions.^8^ Non-reactive P (NRP) in the sludge phase should be firstly converted to soluble-RP (sRP), which could be recovered for direct use. It was known that RP was known as inorganic P (IP), orthophosphate (ortho-P), while NRP was known as organic P (OP) or polyphosphates.^9^ Standards in Measurements and Testing (SMT) protocol was used to investigate the distribution of phosphorus fractional phase in sludge samples.^10^ Thus, the NRP converted to RP fraction was contributed to the anaerobic P release and recovery.

In addition, anaerobic sludge is the pool of microorganisms for P release, which relies on a group of bacteria, *i.e.*, phosphorus accumulating organisms (PAOs).^11^ PAOs could store carbon sources as intracellular polymeric (poly-β-hydroxyalkanoate, PHA) and release ortho-P from the microbial cells using the energy of the degradation of glycogen and polyphosphate (poly-P).^12–14^ PAOs are identified as mesophiles or even psychrophiles.^15^ When the temperature is higher than 20 °C, PAOs and glycogen accumulating organisms (GAOs) will compete for the carbon source uptake. In such a case, GAOs waste the energy, but no polyphosphate will be accumulated for the P recovery.^16^ Moreover, the activity of the phosphatase, which is a kind of hydrolytic enzyme for the P release from organic phosphorus in the sludge, is influenced by the temperature. Therefore, temperature is a key parameter because it affects the microbial population characteristics and the metabolic activity of anaerobic sludge. However, most of the studies focused on the effect of pH on P release of different P fractions, and very few studies were conducted to investigate and identify the effects of temperature on the P fractions and P release.^17^ In this study, a moderate temperature was selected because high temperatures would consume more energy and lead to the lower process stability, especially for the operation of WWTPs in cold regions.^18^ Moreover, it was reported that temperature higher than 20 °C was unfavorable for biological phosphorus removal.^19^ In such a case, a reasonable wider temperature range, 5–35 °C, was considered and investigated for anaerobic treatments in this study. Thus, the low-temperature thermal treatment for phosphorus release was regarded as a low energy process, which led to a biodegradability enhancement and a simultaneous release of P from anaerobic sludge.

In addition to the microbial cells, extracellular polymeric substances (EPS), which store a large amount of phosphorus, is the other important factor in the anaerobic phosphorus release process.^20,21^ EPS is composed of organic compounds such as proteins, carbohydrates, humic acid and phospholipid, which could protect microbial cells and combine with inorganic ions like Ca or Mg. In fact, 27–30% of total P (TP) was contained in EPS in activated sludge.^22,23^ Meanwhile, EPS influenced the transformation of P between bulk solution and PAOs because the sludge flocs were crabbed with EPS matrix and their transformation should be passed through. EPS had great influence on microbial aggregates because of its special characteristics due to its components of EPS, such as polysaccharide, proteins.^24^ The main phosphorus fractions in EPS were identified as the coexistence of ortho-P, poly-P using ^31^P nuclear magnetic resonance (NMR) technology.^25,26^ The phosphorus release of EPS was obviously influenced by many factors, such as organic loading, influent metal concentration and pH.^27,28^ But few studies were conducted to validate the influence of temperature on the P release and P fractions of EPS.

Although the temperature affected the distribution of phosphorus fractions because of their different removal mechanisms and transformation, the effects of temperature on the characteristics and function of phosphorus fractions in intracellular cells and EPS needed to be further investigated.^29^ Thus, it was essential to find out the optimum temperature for EPS production and degradation as well as optimum for P release by groups of bacteria. Facing the aforementioned problems, this study, therefore, explored that how the temperature affected the migration and transformation of phosphorus among microbial cells, EPS and liquid phase using ^31^P-NMR method. The main contributions of this study include (1) to study the effect of temperature on the relationship between phosphorus fractions and anaerobic P release; (2) to investigate the roles of EPS played in the anaerobic NRP fraction to RP fraction conversion process; (3) to identify the optimal temperature for the P release in both intracellular and extracellular phases.

## 2. Materials and methods

### 2.1 Phosphorus release test of anaerobic sludge

The WAS in this study was obtained from the secondary sedimentation tank of a WWTP with an anaerobic/anoxic/aerobic (A^2^/O) process located in Shenzhen, China. The WAS sample was firstly thickened for 24 h at 4 °C by gravitational sedimentation (97% moisture content after thickening), and then screened with a 1 mm sieve and finally stored at 4 °C for the following tests. The main characteristics of activated sludge were as follows: pH 6.6; total suspended solids (TSS) 18.95 g L^−1^, volatile suspended solids (VSS)/TSS ratio of 0.64; soluble chemical oxygen demand (SCOD) 260.5 mg L^−1^; ammonia nitrogen (NH_4_^+^-N) 72.5 mg L^−1^; ortho-P concentration (PO_4_^3−^-P) 38.9 mg L^−1^; soluble Ca 32 mg L^−1^; and soluble Mg 24 mg L^−1^.

In the anaerobic phosphorus release batch laboratory-scale tests, 300 mL sludge samples were placed into 500 mL capped and sealed beakers, which were incubated in a temperature-controlling shaking water-bath (SHZ-B, Xinrui, China) at 5, 15, 25, 35 °C for 200 min (note that the test duration was set as 200 min because the P release kinetic tests could reach their equilibria within 200 min, which was also validated by Xu *et al.*).^3^ Sodium acetate (6.25 mmol L^−1^) was added as the sole carbon source for enhancing the anaerobic phosphorus release. The pH was controlled at 7.0 by adding 1 M HCl or NaOH. The mixed solution (30 mL) was taken out every 20 min for the extraction of phosphorus in sludge (10 mL) and the extraction of EPS (20 mL). The supernatant was measured for the analysis of TP concentration in the bulk solution (TP_L_), while the sludge samples were dried at 105 °C for 24 h. The powdered sludge samples were sieved by passing through 100 mesh sieves to analyze the P fraction in the solid phase.

### 2.2 Phosphorus fractions in sludge samples

The total phosphorus in sludge (TP_s_) was divided into four fractions according to the SMT protocol: organic P (OP_s_), inorganic P (IP_s_), apatite inorganic phosphorus (AP_s_, the P species associated with Ca) and non-apatite inorganic phosphorus (NAIP_s_, the P species associated with oxides and hydroxides of Al, Fe, Mg and Mn), in order to investigate the characteristics and behaviors of phosphorus release. In brief, sludge samples were firstly dried for 48 h and the powdered sludge samples were used for subsequent fractionation. TP_s_ was determined by processing the sludge sample at 450 °C for 3 h following by HCl extraction (3.5 M). IP_s_ was extracted by HCl (1 M), and the residue (OP_s_) was treated at 450 °C for 3 h by HCl extraction (1 M). NAIP_s_ was firstly extracted by NaOH (1 M) and the supernatant was then extracted by HCl (3.5 M), while AP_s_ was extracted by HCl (1 M). The samples were frozen immediately at −80 °C, lyophilized at −20 °C until analysis. Every process needed to follow by stirring for 16 h at room temperature. The phosphorus concentration in the supernatant after extraction processes was measured by the molybdenum blue method as described in Standard Methods.^9^ The detailed extraction processes were followed by the precious publications.^8,26^

### 2.3 Extraction of EPS and collection of microbial cells

Formaldehyde/NaOH (0.06 mL, 36.5% formaldehyde/4 mL, 1 M NaOH) was used to extract EPS matrix from sludge samples at 4 °C according to Liu *et al.*, which was with a high phosphorus extraction efficiency but without the damage of microbial cells.^30^ The EPS extraction was freeze-dried for 48 h at −50 °C for the subsequent ^31^P-NMR analysis.

The centrifugal precipitation (through the 0.2 μm membrane) was regarded as the microbial cell. 0.4 mL of 100 mmol L^−1^ EDTA solution, 0.6 g of 0.1 mm quartz-sand and 3 steel balls were added into the freeze-dried microbial cells. Then, the intracellular phosphorus was largely released from microbial cell samples using the automatic grinding instrument (Tissuelyser-FEII, Jingxin, China) and centrifuged twice at 0 ± 2 °C for 20 min. The centrifugal supernatant was used for total phosphorus concentration analysis and ^31^P-NMR analysis.

### 2.4 ^31^P-NMR analysis

The freeze-dried EPS extraction (15 mg) was dissolved with 0.4 mL of D_2_O and 0.2 mL of 100 mmol L^−1^ EDTA solution. And the microbial cell centrifugal supernatant (0.2 mL) added with 0.4 mL of D_2_O was prepared for the following ^31^P-NMR analysis using an NMR spectrometer (Bruker Avance-600 MHz., Germany) at 162 MHz for ^31^P.^31^ Before analysis, 0.2 mL of 2 mol L^−1^ NaOH needed to be dissolved in D_2_O. The acquisition parameters were as follows: acquisition time, 0.51 s; relaxation delay, 50 s; 90° pulse width, 9.6 μs and an external 85% H_3_PO_4_ standard (*δ* = 0). The spectra were analyzed according to a previous study and the data were determined by NMR data processing software (MestReNova v6.1.0–6224).^32^

### 2.5 Kinetic modeling of TP accumulation in bulk solution

Kinetic models (pseudo-first-order, PFO; pseudo-second-order, PSO) were built to describe the process of phosphorus releasing into the bulk solution. The PFO and PSO models were determined by eqn (1) and (2) and eqn (3) and (4), respectively.^33^1
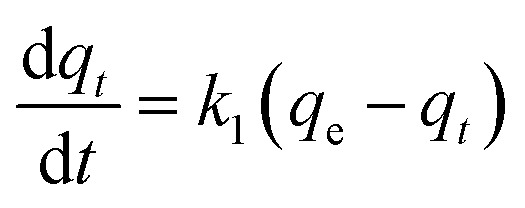

2 ln(*q*_e_ − *q*_*t*_) = ln *q*_e_ − *k*_1_*t*

3
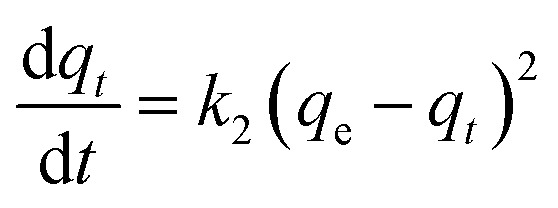
4
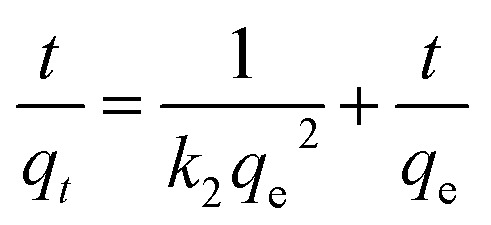
where, *k*_1_ is the rate constant of PFO, min^−1^, *k*_2_ is the rate constant of PSO, L (mg min)^−1^, *q*_*t*_ (mg L^−1^) is the concentration of TP_L_ at time *t* (min), *q*_e_ is the equilibrium TP concentration, mg L^−1^. The correlation coefficient (*R*^2^), normalized standard deviation (Δ*q* (%)) and average relative error (ARE (%)) were used to evaluate the accuracy of these two models, which were determined by eqn (5) and (6):^34^5
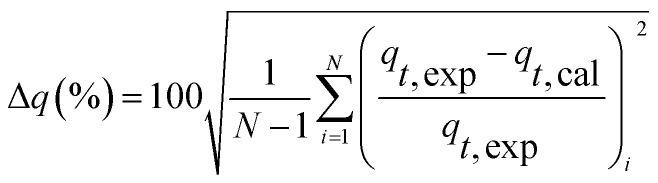
6
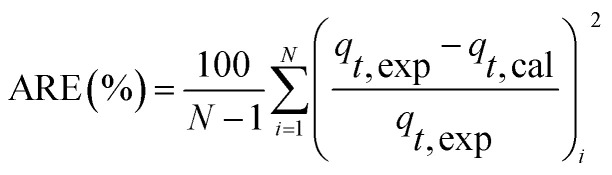
where, *N* is the measurements number, *q*_e,cal_ is the calculated value and *q*_e,exp_ is the experimental value, mg L^−1^.

### 2.6 Other analytical methods

The sludge samples were taken out and immediately centrifuged at 3000 rpm for 10 min (TDL-80-2C, Anting, China) with the total phosphorus (TP) content in the supernatant phase being measured. The TP concentrations in bulk solution (TP_L_), EPS (TP_EPS_) and microbial cell (TP_cell_) were measured using spectrophotometer (T6, Beijing Purkinje General Instrument, Beijing, China) according to the molybdenum blue method in Standard Methods.^9^ Total suspended solids (TSS) and volatile suspended solids (VSS) were analyzed according to the standard method. The acetate concentration was measured by a gas chromatography (7980A, Agilent Technologies, USA) equipped with a flame ionization detector. The measurements of glycogen, PHA intracellular polymers (poly-β-hydroxybutyrate (PHB) and polyhydroxyvalerate (PHV)) and acetate in the anaerobic sludge were performed according to Wang *et al.*^35^ Extracellular proteins in EPS phase were determined with the Lowry method and extracellular polysaccharides were measured by the phenol-sulfuric acid method.^36,37^

## 3. Results and discussion

### 3.1 Phosphorus concentration and distribution in anaerobic sludge at different temperatures

**Fig. 1:**
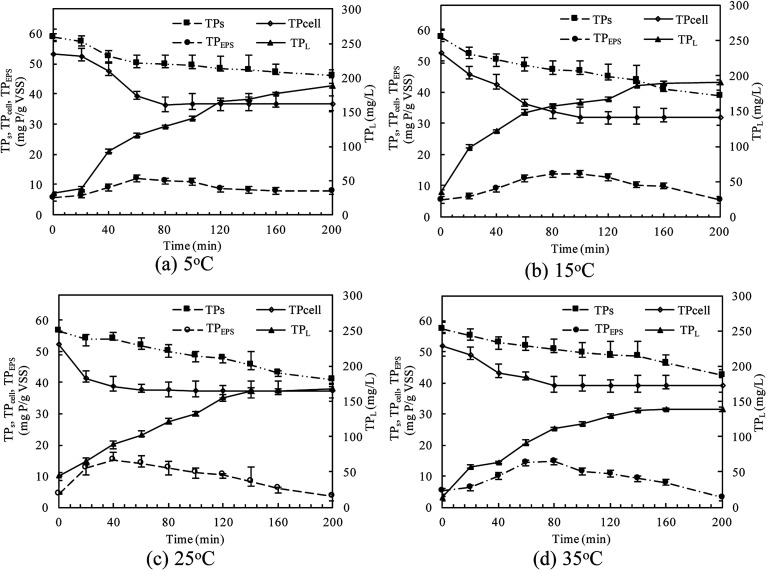
Changes of TP concentration and distribution in anaerobic sludge at different temperatures.

At different temperatures, the TP concentration and distribution during the anaerobic treatments were shown in Fig. 1. The concentration of TP_L_ rapidly increased with a decrease of TP_s_ concentration, indicating that phosphorus in the sludge phase was released into the bulk solution during the anaerobic treatment. The highest TP_L_ concentration reached 190.2 mg L^−1^ at 15 °C, while, at other temperatures, the sludge exhibited a relatively lower P release ability. The changes of TP_L_ concentration at different temperatures were mainly caused by two reasons: the potential release of sludge and the hydrolysis of EPS-associated phosphorus.

**Fig. 2:**
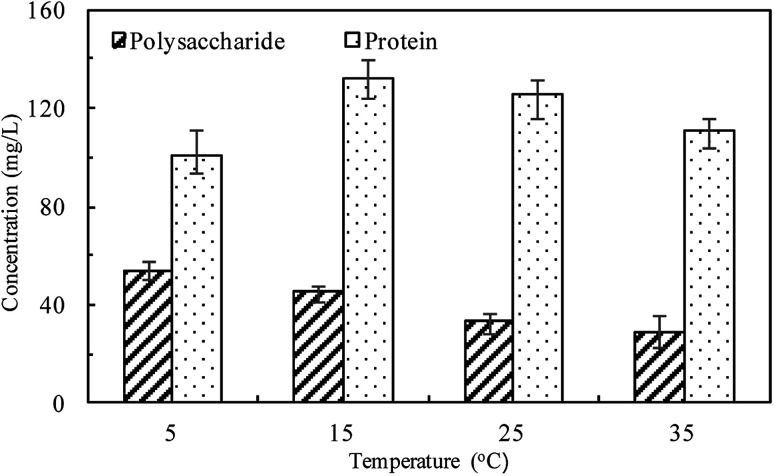
Changes of EPS components in anaerobic sludge at different temperatures.

The TP contents of anaerobic sludge released phosphorus at 5 °C, 15 °C, 25 °C and 35 °C were 12.85 mg g_VSS_^−1^, 18.97 mg g_VSS_^−1^, 15.56 mg g_VSS_^−1^ and 14.86 mg g_VSS_^−1^, respectively. The content of TP_cell_ showed a similar trend with TP_s_ concentration, and the extremely low temperature (5 °C) may be harmful to the activities of PAO cells in storing P as intracellular poly-P. The content of TP_cell_ kept almost constant at first 20 min for adapting to the harsh environment and then decreased rapidly at 5 °C. EPS was mainly comprised of protein and polysaccharide. As shown in Fig. 2, temperature also obviously affected the EPS components, especially the polysaccharide content that was increased by a low temperature. Liu *et al.* similarly concluded that the polysaccharides content of EPS at 5 °C was two-fold higher than those at 15 and 25 °C.^38^ The increase of polysaccharide content improved the adsorption ability of EPS and hence the P accumulating as well as microbial aggregation in the EPS phase.^39^ EPS showed a stronger hydrophilicity at a lower temperature. Due to the protection of protein tightly packed around the sludge flocs, some metal ions could be precipitated with P entrapped in the EPS matrix and affected the P release.^40^ After 200 min-anaerobic phosphorus releasing process, 2.21 mg g_VSS_^−1^ of TP_EPS_ was released at 35 °C, but only 0.17 mg g_VSS_^−1^ and 0.75 mg g_VSS_^−1^ of TP_EPS_ were absorbed by EPS at 15 °C and 25 °C, respectively. In addition, 2.18 mg g_VSS_^−1^ of phosphorus was accumulated in the EPS at 5 °C, which demonstrated that a higher content of polysaccharide in EPS enhanced the P accumulation in EPS. Notably, the TP_EPS_ content approached balance in the later period of the anaerobic process at 5 °C (Fig. 1), but a continuous decline occurred at other temperature tests. This may be because the extremely low temperature was not beneficial for the P transformation from microbial cells and the hydrolysis of EPS slowed down caused by few exopolyphosphatases in EPS. At 35 °C, TP_EPS_ gradually increased from 5.18 mg g_VSS_^−1^ to 14.67 mg g_VSS_^−1^ at 60 min, which showed an obvious P accumulation.^41^ Then, TP_EPS_ significantly decreased to 2.97 mg g_VSS_^−1^ because of the EPS hydrolysis, which could reduce macromolecules and particulate matter into the metabolizable low-molecular-weight P. Thus, the contribution of extracellular P release could not be neglected.

**Fig. 3:**
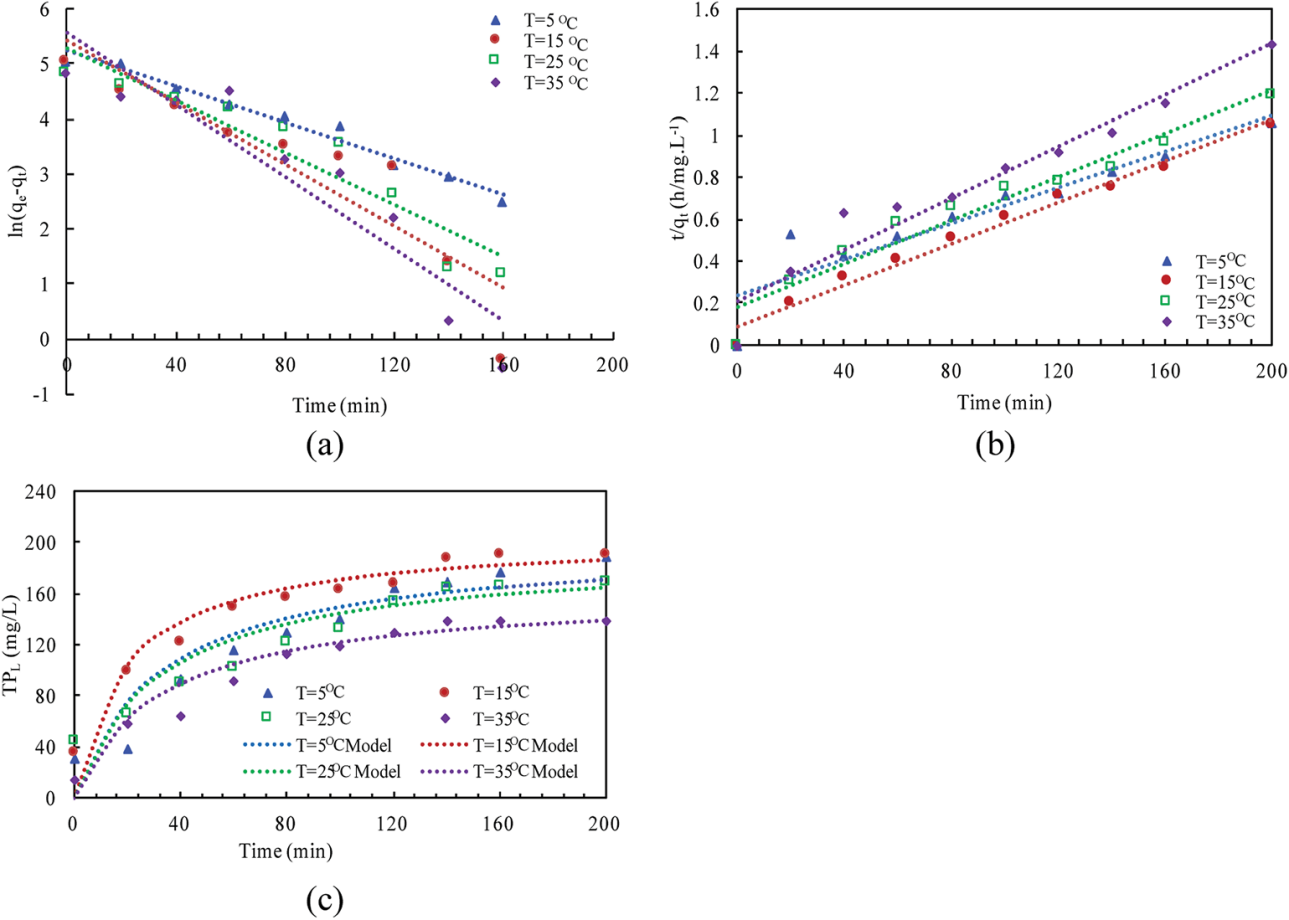
Kinetic modeling of TP_L_ release in the liquid phase at different temperatures (a) fitting line of pseudo-first-order; (b) fitting line of pseudo-second-order; (c) match curves between measured and modeled values in pseudo-second-order model.

The linear expressions of the two models were presented in Fig. 3 and the parameters were presented in Table 1. The kinetic models were used to understand the phosphorus release to the bulk solution. According to the *R*^2^ values, the identified PSO model was better than PFO model for phosphorus accumulation in the bulk solution. The model parameter *k*_2_ was highest at 15 °C with the largest amount of *q*_e_ (204.08 mg L^−1^), indicating that 15 °C was the possible best temperature for the anaerobic phosphorus release. And the initial release rates expressed as *k*_2_*q*_e_^2^, were 6.002, 10.412, 5.917 and 4.94 mg (L min)^−1^ along with the temperature increased from 5 °C to 35 °C. Thus, the phosphorus release from the solid to liquid phase was influenced by the pseudo-second-order reaction at different temperatures and the low temperature (especially 15 °C) was preferable for P release into the bulk solution.

**Table 1:** Kinetic parameters of two models at different temperatures

		Temperature (°C)			
		5	15	25	35
Pseudo-first-order	*k*_1_ (min^−1^)	0.0237	0.028	0.0362	0.0327
	*q*_e,cal_ (mg L^−1^)	215.21	223.97	322.95	259.08
	*R* ^2^	0.8895	0.8356	0.8069	0.8731
Pseudo-second-order	*k*_2_ (L (mg min)^−1^)	0.00015	0.00025	0.00016	0.00019
	*q*_e,cal_ (mg L^−1^)	200.03	204.08	192.30	161.29
	*R* ^2^	0.9522	0.9822	0.9464	0.9409
	Δ*q* (%)	7.63	8.93	7.55	10.07
	ARE (%)	0.58	0.79	1.02	1.01

### 3.2 Phosphorus fractions of anaerobic sludge by SMT protocol

**Fig. 4:**
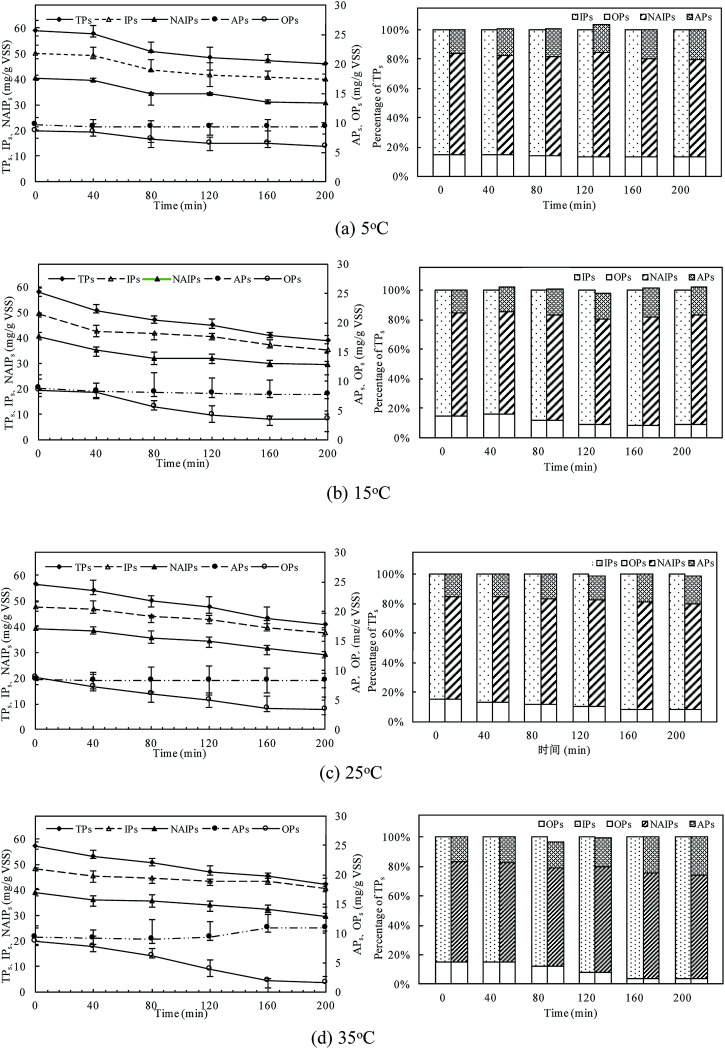
Changes of phosphorus fractions in the sludge phase at different temperatures.

The changes of phosphorus fractions and distribution in the sludge phase at different temperatures were shown in Fig. 4. P mass stored in the residue sludge could be used as an indicator to assess the P release ability of PAOs. The P release amount was obviously affected by the temperature, with TP_s_ release percentages of 21.8%, 32.8%, 27.6% and 25.9% with the temperature of 5 °C, 15 °C, 25 °C, and 35 °C, respectively. The TP_s_ was the sum of IP_s_ and OP_s_, and its content decreased to a minimum value of 31.58 mg g_VSS_^−1^ at 15 °C. The consistent result was obtained with that presented in the above session (Section 3.1).

IP_s_ was the major P fraction of anaerobic sludge and mainly dominated P dynamics due to the high content of the labile P fraction (NAIP_s_). NAIP_s_ accounted for more than 75% of IP_s_ and the percentage of NAIP_s_ kept constant with the increase in temperature. The release efficiencies of IP_s_ were related to the changes of NAIP_s_ and AP_s_. The NAIP_s_ was considered as the most labile phosphorus fraction and its release would be enhanced by the increase of temperature. The content of AP_s_ was almost unchanged at all temperatures indicating that AP_s_ was much more stable than NAIP_s_. At 35 °C, the ratio of AP_s_/IP_s_ increased obviously with a relative decrease of NAIP_s_/IP_s_, indicating that a higher temperature could effectively improve the P bioavailability resulting from the conversion of NAIP_s_ to AP_s_.

Moreover, the proportion of OP_s_, named as one of the main compounds of NRP in TP_s_, was relatively stable in the all temperatures conditions. But the release rate of OP_s_ at higher temperature was much quicker than that at low temperatures, indicating that a higher temperature could accelerate the release of OP_s_ with the dissolution of IP_s_ combined with metal ions in the sludge phase. The content of OP_s_ was almost unchanged at the first 40 min of the anaerobic process, and then significantly decreased. The release content of OP_s_ reached the maximum value of 7.04 mg g_VSS_^−1^ at 35 °C, while only 2.63 mg g_VSS_^−1^ of OP_s_ was released at 5 °C. At 35 °C, P amount stored in the biomass decreased by 80.73% of OP_s_ after 200 min anaerobic process, but the OP_s_ release was tended to be stable due to the inhibition of PAOs activity. The release of OP_s_ increased with temperature increasing due to both the sludge disintegration and microbial metabolism.

Thus, different P fractions and compositions behave differently as IP_s_ could be easier to release, while OP_s_ commonly passed through. The sum of NAIP_s_ and OP_s_ was regarded as potential bio-available phosphorus for the following phosphorus release.^42^ The percentage of NAIP_s_ and OP_s_ in this study was about 78.01–82.24%, and the ratio of potential bio-available phosphorus to TP_s_ kept almost constant during the anaerobic process, thus, it showed a high potentially bio-available phosphorus stored in the anaerobic sludge.

### 3.3 Phosphorus fractions of EPS

Because anaerobic processes at lower temperatures destroyed the polymeric structure of EPS and strengthened the attached components of microbial cells, macromolecular polyphosphates released from microbial cells could not release into the supernatant directly. Phosphorus released from microbial cells firstly leaked into the EPS and then desorbed the most of EPS-associated P into the bulk solution.

**Fig. 5:**
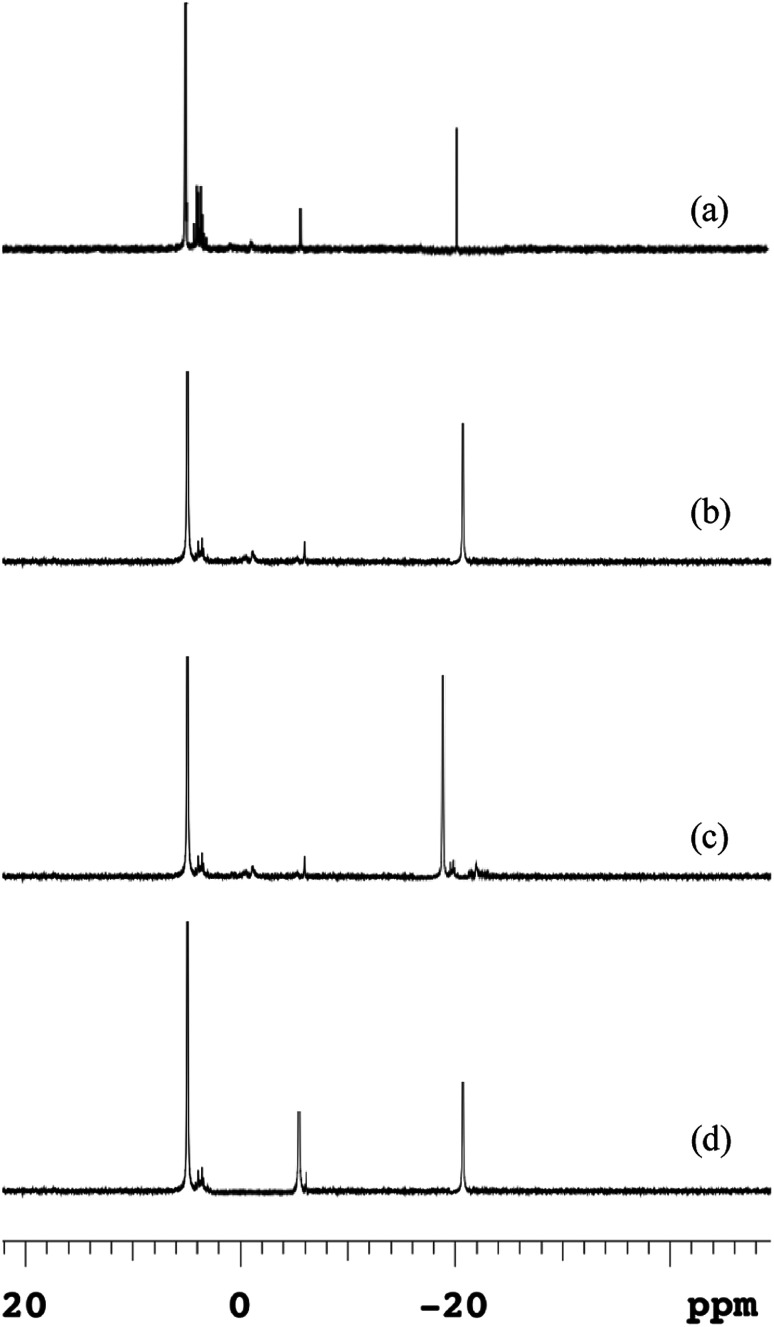
Changes of ^31^P-NMR spectra of EPS in anaerobic sludge at different temperatures (a) 5 °C; (b) 15 °C; (c) 25 °C; (d) 35 °C.

The ^31^P-NMR spectroscopies of EPS at different temperatures were shown in Fig. 5, and the chemical shifts and relative percentage of identified phosphorus fractions were presented in Table S1.† Five P species, *i.e.*, ortho-P, poly-P, orthophosphate monoesters (monoester-P), orthophosphate diesters (diester-P) and pyrophosphate (pyro-P), were identified in EPS from their signals at 5.48, –4.37 for end groups of poly-P, –18.89 for middle groups of poly-P, 3.78–4.16, –2.05 and –5.01 ppm, respectively. The ^31^P-NMR spectroscopy showed that poly-P and ortho-P were the dominant forms of P in EPS. The signals of organic phosphorus fractions (monoester-P and diester-P) were observed at 5 °C but the signals of organic P almost disappeared and those of inorganic P remained at other temperatures. The monoester-P and diester-P, related to the activity and concentration of living cells, were released with the disintegration of sludge and then transformed to inorganic phosphorus. Such complex P components in the EPS at 5 °C revealed that the activity of degrading enzymes (exopolyphosphatases) in the hydrolysis of EPS was weakened at a low temperature. This observation was similar with the release trend in the anaerobic sludge (Fig. 4), and a higher temperature was beneficial to transform the OP_s_ to IP_s_ (especially NAIP_s_), which led to a conversion to the RP fraction.

The poly-P in the raw sludge phase was long-chain (about 42) by ^31^P-NMR spectra, which was reduced during the anaerobic treatment. The similar result was reported by Lichko *et al.*^43^ The mean chain length of poly-P was calculated as eqn (7). The chain length of poly-P at moderate temperatures was much shorter than that at a lower temperature. This indicated that the hydrolysis of EPS-associated poly-P was enhanced by higher temperatures with the degradation of the long-chain poly-P. The long-chain poly-P intercepted by EPS was further degraded to short-chain poly-P and pyro-P by PAOs. These short-chain poly-P and pyro-P were then degraded to ortho-P and released into the liquid. As the amounts of EPS increased at lower temperatures, more macromolecular phosphorus was accumulated in EPS. Lower temperatures led to a much stronger affinity with multi-chain phosphorus. But the extracellular enzyme was not enough to provide the energy for the hydrolysis of EPS in a very low-temperature condition (5 °C). Thus, the proportion of poly-P was much higher than those at other temperatures (reflected by higher peak contents). The degradation of high molecular weight poly-P and pyro-P associated with polymeric materials could provide the energy for the P release process.

7 Poly-P mean chain length = 2 + 2 (middle groups of poly-P/end groups of poly-P)

Similarly, ortho-P was easily found in nucleic acids or hydroxide groups in polysaccharide of EPS, because the content of polysaccharide in EPS was significantly increased at the lower temperature.^44^ Ortho-P existed in EPS was related to two sources, *i.e.*, intracellular poly-P directly leaked into the extracellular polymeric matrix and extracellular poly-P phase degraded to ortho-P by degrading enzymes.

The binding between EPS-associated P (negative charges) and metal ions, such as Ca^2+^, Mg^2+^ was enhanced by lower temperatures, which was the main nutrient for living organisms.^25^ These two-divalent cation-P compounds named as particular reactive P (pRP) were presented in the EPS matrix. Such intermolecular interactions were the basis of maintaining the microbial aggregate structure with a higher proportion of ortho-P (soluble RP, sRP) in EPS.^8^ And extracellular ortho-P and its precipitates in biological phosphorus accumulation were dominant in EPS. Such results were similar with the observation of Cloete and Oosthuizen, who found that phosphate precipitation was absorbed by EPS in the WWTPs.^23^

### 3.4 Intracellular phosphorus contents

Temperature is the key parameter affecting the microbial population and organic components during the anaerobic phosphorus release process, as shown in Table 2 and Fig. S1.† The concentrations of the soluble ortho-P (PO_4_^3−^-P) and acetate during the anaerobic release process were consistent with the expected enriched PAOs. A large amount of organic P and polyphosphates was accumulated in the microbial cellular materials, and the intracellular release process could be realized a conversion from NRP to sRP fraction by PAOs.

**Table 2:** Biomass composition of anaerobic sludge at different temperatures^a^

Parameter	5 °C	15 °C	25 °C	35 °C
MLSS (mg L^−1^)	2500	2320	2420	2030
MLVSS (mg L^−1^)	2240	2130	1870	1680
Poly-P (mg L^−1^)	142.11	77.89	451.58	261.58
Active biomass (mg L^−1^)	1896.71	1768.05	1509.54	1415.31
P_release_/HAc_uptake_ (P-mol/C-mol)	0.68	0.75	0.53	0.22
Glycogen_degraded_/HAc_uptake_ (C-mol/C-mol)	0.50	0.49	0.99	1.03
PHA_synthesized_/HAc_uptake_ (C-mol/C-mol)	1.15	1.04	1.01	1.35
PHV/PHB	0.05	0.02	0.05	0.16

a Ash = MLSS-MLVSS; poly-P = Ash-MLVSS × 5/95; active biomass = MLVSS-PHA-Gly.^48^

The metabolism of PAOs was dependent on the internal stored carbon compound and energy source. The glycogen concentration at the first 10 min was not decreased obviously with an obvious increase of phosphorus concentration in the liquid phase, which verified that poly-P in microbial cells may be used as the prior energy for the anaerobic P release with the long-chain poly-P degraded to short-chain poly-P or ortho-P. The intracellular poly-P storage at 15 °C was the least while the hydrolysis of intracellular poly-P was limited when temperature increased, indicating that the microbial population of anaerobic sludge with a lower intracellular P-content at other temperatures might consist of species other than PAOs (*i.e.*, GAOs). The presence of GAOs might interfere with the accumulation of poly-P by PAOs.^11^

The PHA compounds contained PHB and PHV, and their values increased with the release proceeding. The increased PHA content accelerated the disintegration and the following cell lysis of anaerobic sludge. Moreover, more PHV formed in the later period of anaerobic treatments especially at higher temperatures due to more glycogen degradation.^44^ This phenomenon could be explained by the reason that the glycogen pathways gradually replaced the poly-P pathways for P release.

The higher PHA_synthesized_/HAc_uptake_ ratio occurred at 35 °C, but a higher P-release did not occur under this temperature condition. The ratio of P_release_/HAc_uptake_ reduced from 0.75 P-mol/C-mol (15 °C) to 0.22 P-mol/C-mol (35 °C), indicating the metabolic pathway of PAOs shifted polyphosphate-accumulating metabolism (PAM) to glycogen-accumulating metabolism (GAM) when the temperature increased from 15 °C. And the ratio of Glycogen_degraded_/HAc_uptake_ was in a range of 0.31–0.50 C-mol/C-mol for the PAO-enriched cultures, while 0.92–1.25 C-mol/C-mol for the GAO-enriched cultures, the stoichiometric ratio of Glycogen_degraded_/HAc_uptake_ in this study indicated that the metabolic pathway of PAOs was shifted to GAM at higher temperatures (25 °C and 35 °C).^45^ The similar results have been reported by Mulkerrins *et al.*^46^ Thus, the microbial population dynamics showed a shift from the dominance of PAOs to GAOs with the increase of temperature. Notably, the phosphorus concentration in the bulk solution was much lower than that at other temperatures, while the release content of TP_EPS_ at 35 °C was largest. This was verified that P release processes between extracellular and intercellular substances were independent. Because the intracellular poly-P was limited at 35 °C, the glycogen utilization might be gradually replaced by the tricarboxylic acid (TCA). PAOs could tune their metabolic pathway between the dominance of PAM and GAM along with the change of intracellular available poly-P level.^47^

Although the content of active biomass was highest at 5 °C (MLVSS content = 2240 mg L^−1^), the intracellular poly-P content was higher than that at 15 °C. This was because the activity of exopolyphosphatases was weakened at the relatively low temperature, and hence some short-chain poly-P could not be degraded into ortho-P. As a result, they could be stored in the EPS matrix and not participated in the following P release. That's why the concentration of TP_EPS_ at 5 °C after 200 min-anaerobic P release was increased.

### 3.5 Relationship between intracellular and extracellular P release/transfer

The P release ability of anaerobic sludge was dependent on both intracellular and extracellular P release processes. The P release processes in intracellular and extracellular phases were relational and relatively independent with each other. Temperature could not only change the release of different P fractions in the sludge phase, but also change the mechanisms of PAOs and EPS reaction process.

Based on the above results, a possible organically-bound P-release pathway of anaerobic sludge at different temperatures was proposed to enhance P-release with the increase of TP_L_ concentration in the bulk solution. Firstly, EPS not only transported ortho-P into the bulk solution, but also stored P (short-chain poly-P and ortho-P) in EPS. Long *et al.* also verified that EPS played an important role in transfer and transformation of ortho-P during the anaerobic P release.^25^ Due to the high bio-sorption ability, the more proportion of extracellular phosphate minerals was accumulated in EPS at a low temperature (2.18 mg g_VSS_^−1^ at 5 °C). Secondly, PAOs were found to changing their metabolic pathways from PAM to GAM with the increase of temperature. PAOs in the anaerobic sludge could preferentially utilize the energy from the degradation of intracellular poly-P (especially long-chain poly-P) for metabolic activity for P release. The autolysis of microbial cells was the driving force for the release of intracellular poly-P. The hydrolysis of poly-P led to the final release of ortho-P.^49,50^ Li *et al.* verified that the highest PAOs activity was obtained at a moderate temperature.^51^ But the mass transfer between microbial cells and EPS was not benefited by 5 °C because bacteria in microbial aggregates was protected by more content of EPS in “uncomfortable” environment for survival. Therefore, intracellular P dynamic played a decisive role for the anaerobic P release at different temperatures.

## 4. Conclusions

The study investigated the effect of temperature varied from 5 °C to 35 °C on the anaerobic P release. The interactions between the role of EPS and the PAOs metabolic pathway for the conversation from NRP to RP fraction and the anaerobic P release were considered. The main conclusions are summarized as follows:

(1) The intracellular biochemical metabolism and EPS-associated P reactions, *i.e.*, hydrolysis and adsorption processes, were relatively independent. In this study, 15 °C was the optimal temperature for the conversion from NRP to RP fraction in the sludge phase due to the highest TP_L_ concentration observed.

(2) A higher temperature could effectively improve the P bioavailability resulting from the conversion of NAIP_s_ to AP_s_. The ratio of potential bio-available phosphorus to TP_s_ kept almost constant (78.01–82.24%), because of a high potential bio-available phosphorus stored in the anaerobic sludge.

(3) Poly-P and ortho-P were the dominant forms of P in EPS. A higher temperature enhanced the hydrolysis of EPS, which reduced the macromolecules and particulate matter into the metabolizable low-molecular-weight P.

(4) P stored in EPS and transported by EPS were closely related to the PAOs activity and the intracellular P dynamic metabolism played a decisive role for P release.

## Conflicts of interest

There are no conflicts to declare.

## Supplementary Material

RA-009-C8RA10048A-s001
