# Supplementary material for: Temperature effect on extracellular polymeric substances (EPS) and phosphorus accumulating organisms (PAOs) for phosphorus release of anaerobic sludge
Source: RSC Adv. 2019 Jan 16;9(4):2162–71. doi: 10.1039/c8ra10048a (PMC9059762; doi:10.1039/c8ra10048a)
Supplement: RA-009-C8RA10048A-s001 [file RA-009-C8RA10048A-s001.pdf]

Electronic Supplementary Material (ESI) for RSC Advances.
This journal is © The Royal Society of Chemistry 2019

Supplementary materials

Table S1 Chemical shifts and relatively peak areas of different-species phosphorus in the ^{31}P-NMR spectra at different temperatures

	Ortho-P		Monoesters-P		Diesters-P		Pyro-P	
	Chemical shift (ppm)	Peak area (%)	Chemical shift (ppm)	Peak area (%)	Chemical shift (ppm)	Peak area (%)	Chemical shift (ppm)	Peak area (%)
5°C	5.68	42.3	4.16	3.4	-2.05	1.3	-5.01	3.5
15°C	5.48	45.05	-	-	-	-	-5.01	2.48
25°C	5.71	55.04	-	-	-	-	-5.01	14.23
35°C	5.48	30.2	-	-	-	-	-5.01	3.6

	End groups of poly-P		Middle groups of poly-P		Average chain length of poly-P
	Chemical shift (ppm)	Peak area (%)	Chemical shift (ppm)	Peak area (%)	
5°C	-4.37	10.3	-19.38<br>-20.90	39.2	9.6
15°C	-4.51<br>-4.57	5.67	-18.89<br>-18.93<br>-19.53<br>-20.90	46.8	18.51
25°C	-4.37<br>-4.51	6.86	-18.53<br>-18.89<br>-18.93	23.87	8.96
35°C	-4.46<br>-4.53	20.6		45.6	6.43

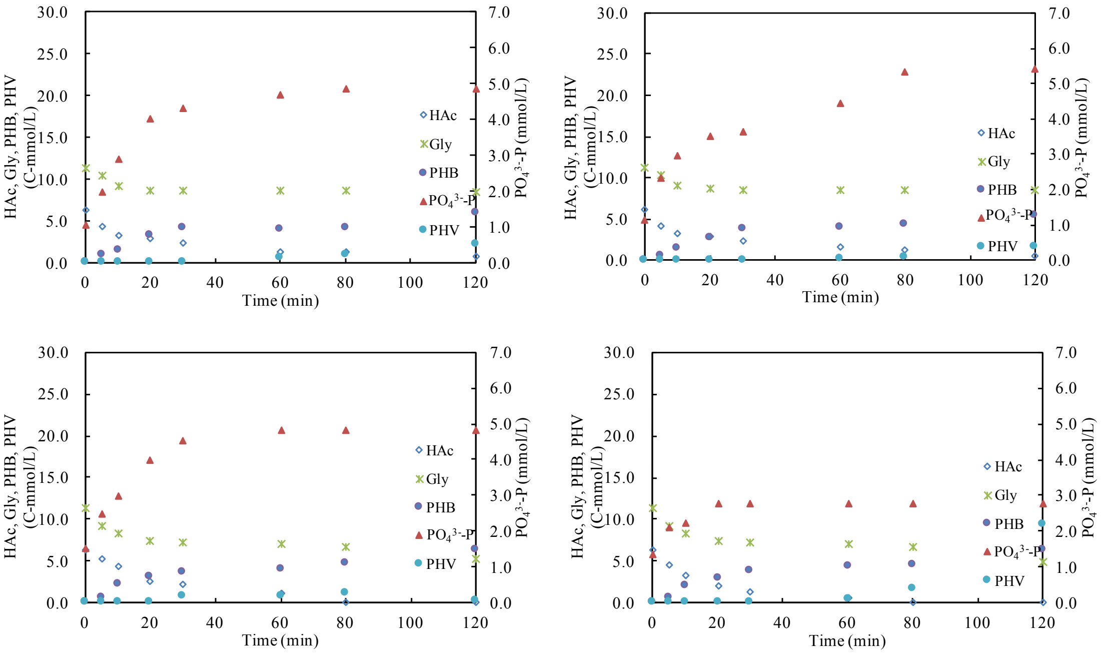

Fig. S1 Changes of TP concentration and distribution in anaerobic sludge at different temperatures
